# Species Composition, Diversity, Abundance, and Phylogeny of Fleas of Small Mammals in Selected Plague-Endemic and Nonendemic Areas of Tanzania

**DOI:** 10.1155/japr/5513199

**Published:** 2025-11-21

**Authors:** Adrian E. Materu, Eliakunda M. Mafie, Jahashi S. Nzalawahe, Ladslaus L. Mnyone

**Affiliations:** ^1^Department of Microbiology, Parasitology and Biotechnology, College of Veterinary Medicine and Biomedical Sciences, Sokoine University of Agriculture, Morogoro, Tanzania; ^2^Africa Centre of Excellence for Innovative Rodent Pest Management and Biosensor Technology Development, Institute of Pest Management, Sokoine University of Agriculture, Morogoro, Tanzania; ^3^Division of Science, Technology and Innovation, Ministry of Education, Science and Technology, Dodoma, Tanzania

**Keywords:** abundance, diversity, fleas, phylogeny, rodents, shrews

## Abstract

The remerging nature of plague requires detailed understanding of the plague flea vectors and small mammal reservoir interactions. Therefore, this study is aimed at assessing flea vector composition, diversity, prevalence, abundance, and phylogeny in selected villages of Lushoto, Mbulu, and Morogoro Rural districts in Tanzania. Small mammals were captured from households and different habitats, and flea ectoparasites were collected, processed, and identified morphologically. Furthermore, seven specimens of the most collected flea species *Ctenophthalmu*s sp. and *Xenopsylla cheopis* were submitted for advanced molecular identification and phylogenetic relatedness. The prevalence between predictors such as habitat type, host species, host sex, and locality was compared using chi-square tests, and also, generalized linear models (GLMs) were used to check the variation between flea abundance and different predictors. A total of 302 small mammals were captured with *Mastomys natalensis* (*n* = 163, 54.0%), *Rattus rattus* (*n* = 41, 13.6%), and *Crocidura* spp. (*n* = 31, 10.3%) dominating the total capture. The collected fleas belonged to nine species, and the most prevalent and abundant species were *Ctenophthalmus* spp. (*n* = 84, 31.0%), *Pulex irritans* (*n* = 82, 30.3%), *Dinopsylla lypusus* (*n* = 78, 28.8%), and *Nosopsyllus incisus* (*n* = 11, 4.1%) from rodents, and more so on males than females. The highest flea diversity was in crop fields (*H*′ = 1.05) followed by near-natural forests (*H*′ = 1.03) and fallow land (*H*′ = 0.7). Phylogenetic analysis of ITS1 sequences for *Ctenophthalmus* sp. and *Xenopsylla cheopis* from Lushoto and Mbulu districts showed strong nucleotide identity. These findings highlight the need for continuous flea and rodent surveillance to mitigate potential plague outbreaks and protect public health in endemic areas.

**Summary**

• Fleas are small wingless hematophagous insects commonly infesting small mammals; they play an exemplary role in transmitting various pathogenic agents such as bacteria, viruses, protozoa, and helminths. Fleas are among the vectors of the plague bacterium *Yersinia pestis*.
• Knowledge of relationships between flea species and small mammals is very crucial for developing sustainable plague control strategies and prevention.
• The present study investigated the flea species composition, diversity, prevalence, abundance, and phylogeny in small mammals collected in Lushoto, Mbulu, and Morogoro Rural districts in Tanzania. The collected fleas belonged to nine species and the most prevalent and abundant species were *Ctenophthalmus* sp. (*n* = 84, 31.0%), *Pulex irritans* (*n* = 82, 30.3%), *Dinopsylla lypusus* (*n* = 78, 28.8%), and *Nosopsyllus incisus* (*n* = 11, 4.1%) in crop fields and fallow land, and more so in male rodents than females.
• Flea species were more prevalent and abundant on *Mastomys natalensis* (*n* = 108, 39.9%), followed by *Rattus rattus* (*n* = 74, 27.3%), irrespective of districts. The highest flea diversity was in crop fields followed by near-natural forests and fallow land.
• Phylogenetic analysis of ITS1 sequences for *Ctenophthalmus* spp. and *Xenopsylla cheopis* from Lushoto and Mbulu districts showed strong nucleotide identity. These findings highlight variation in terms of flea diversity, prevalence, and abundance within habitats, host species, sex, and locality.

## 1. Introduction

Fleas (order Siphonaptera) are hematophagous insects capable of transmitting various zoonotic agents, including bacteria, viruses, protozoa, and helminths [1–3]. Globally, over 2500 flea species across 16 families and 246 genera have been described [4–7], with most infesting small mammals, particularly rodents [6–9]. Adult fleas may reside on or off the host, depending on their biology, and this behavior varies by taxon, influencing disease transmission dynamics [10, 11]. Fleas also differ in host specificity, ranging from host specific (≤ 2 host species) to host opportunistic (> 2 host species) [5, 10, 12].

Plague, caused by the bacterium *Yersinia pestis*, is one of the most significant flea-borne zoonoses globally, with over 90% of cases and deaths occurring in Africa, including Tanzania [13–16]. Fleas are key to maintaining both enzootic and epizootic plague cycles in endemic regions [11, 17, 18]. Major vector species include *Xenopsylla cheopis*, *Xenopsylla brasiliensis*, *Dinopsylla lypusus*, and *Ctenophthalmus* sp. [16, 19, 20] with *X. cheopis* and *X. brasiliensis* being the most predominant in many African countries, including Tanzania. Small mammals, particularly rodents, serve as primary plague reservoirs, with species like *Mastomys natalensis*, *Rattus rattus*, *Lophuromys* spp., and *Praomys* spp. playing key roles in previous outbreaks [20–23].

Given the re-emerging nature of plague, continuous monitoring of biotic and abiotic factors is essential for outbreak preparedness and management. These factors vary across regions; endemic areas often support conditions such as moderate temperatures, high humidity, dense rodent populations, and abundant vegetation that sustain the plague cycle, while nonendemic areas typically lack such favorable environments for flea vectors and their hosts, resulting in lower disease incidence [24, 25]. Interestingly, even neighboring villages with similar environmental and demographic conditions may differ in plague occurrence as observed in plague foci within Lushoto District, Tanzania [26]. This highlights the complex and localized nature of plague ecology.

Environmental factors such as temperature and humidity significantly influence flea infestations on small mammals, affecting both hosts and parasites directly or indirectly [27, 28]. Seasonal variation also plays a role, with higher host and flea abundance often recorded during dry seasons [29–31]. Habitat modifications—driven by environmental conditions—can impact both fleas and their hosts [28, 32, 33]. Since certain flea developmental stages occur in the environment, their survival depends on optimal temperature and humidity, especially for larvae [27, 31, 34, 35]. Additionally, demographic factors such as human land use can alter host distribution and susceptibility, influencing flea populations [28, 36, 37]. For instance, habitat disturbances and practices like the use of acaricides on livestock may reduce flea survival and infestation rates [28].

Host-associated factors such as age, sex, body size, condition, and immunity significantly influence flea infestation [10, 32, 35, 38, 39]. Sex-biased parasitism often favors males [40–42], while both young and old individuals tend to be more susceptible [43, 44]. Although some studies report male-biased infestation [38, 45], others show mixed patterns, including no bias or rare female bias [46]. Larger hosts offer more surface area and often harbor more fleas, and individuals with poor body condition or weaker immunity are more prone to ectoparasite loads [35, 40, 47]. Additional biotic factors such as sociality, longevity, reproductive status, and grooming behavior also influence infestation [28, 40, 48]. Social species that share nests promote ectoparasite transmission, while long-lived hosts may accumulate more fleas over time. Strong grooming behavior, however, can reduce flea burden [48].

Flea ecology shaped by ecological and host-related factors directly influences the transmission dynamics of plague and other flea-borne diseases. Variations in species composition or increased flea abundance can heighten transmission risk [23, 32, 41]. In Tanzania, plague remains endemic in regions such as Lushoto, Mbulu, and Karatu, where periodic outbreaks have been reported, while other areas remain nonendemic with limited or no recent cases [49]. Understanding host–parasite interactions is vital for effective surveillance and control, while molecular and phylogenetic tools enhance species identification and epidemiological insight. This study is aimed at assessing flea species composition, diversity, prevalence, abundance, and phylogeny in selected plague-endemic and nonendemic regions of Tanzania.

## 2. Materials and Methods

### 2.1. Study Areas

This study was conducted in selected wards and villages of Lushoto District in Tanga Region (endemic) and Mbulu District in Manyara Region (endemic) as well as Morogoro District Council in Morogoro Region (non-endemic). Lushoto District is located between 4° 32⁣′ S and 38° 37⁣′ E and lies mostly within the Western Usambara Mountains which constitute one of the major plague-endemic foci in Tanzania [16]. The study area experiences a wet season from November to May and a dry season from May to November, with annual rainfall ranging from 800 to 2000 mm and an average temperature of 20°C. Mbulu District is located between 03° 51⁣′ S and 35° 32⁣′ E and constitutes another main plague active focus in Tanzania [23, 50]. The study area experiences a wet season from November to May and a dry season from May to November, with an annual rainfall range of 800–1000 mm and an annual temperature range of 20°C–25°C.

Morogoro District Council is located between 06° 54⁣′ S and 37° 54⁣′ E. The study area experiences a wet season from November to May and a dry season from May to November, with an annual rainfall range of 500–2200 mm and a temperature range of 18°C–24°C.

**Figure 1:**
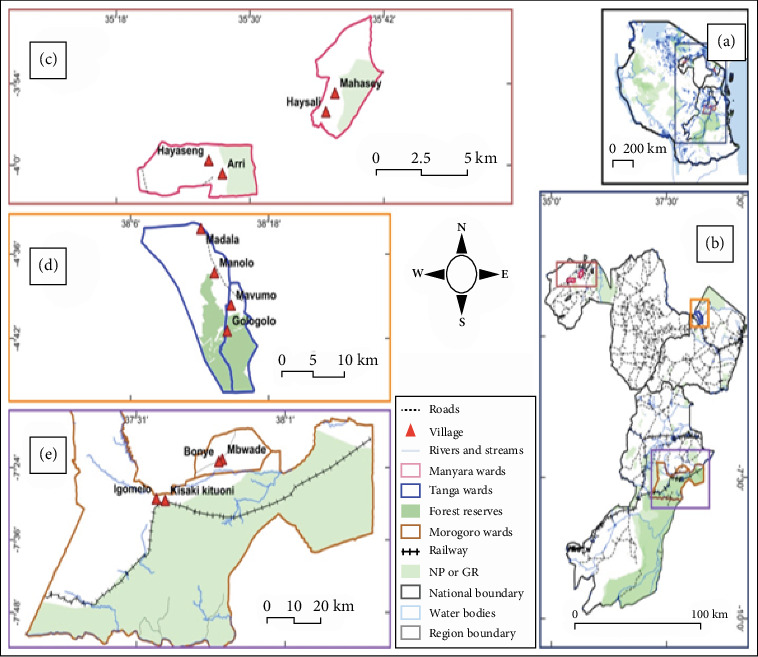
(a) Map of Tanzania showing the selected three study regions. (b) The selected wards in each region. Villages in which the study was conducted are shown by red triangles for wards of (c) Mbulu District, (d) Lushoto District, and (e) Morogoro District Council in the insert.

Two wards and two villages in each district were selected and sampled. Study villages in endemic districts were selected, among other factors, based on previous history of plague outbreaks and likelihood of high rodent population. In nonendemic districts, the study villages were selected based on convenience and likelihood of high rodent population. The study villages in Lushoto District were Mavuno and Gologolo at Shume ward, as well as Madala and Manolo at Manolo ward. The study villages in Mbulu District were Haysali and Nahasey at Nahasey ward as well as Hayaseng and Arri at Yaeda Ampa ward. The study villages in Morogoro District Council were, namely, Kisaki kituoni and Igomelo, Kisaki ward, as well as Bonye and Mbwade, Bwakila Chini ward (Figure 1).

### 2.2. Study Design

The study employed a cross-sectional design, and each study village was sampled once for three consecutive nights during the period from March to June 2022. The sampling of rodents and associated ectoparasites was conducted inside the house (five in each village), fallow land, crop fields, plantation forests, near-natural forest, and bushes. The study habitats can be defined as follows: fallow land, previously cultivated areas left unplanted, allowing natural vegetation such as grasses and shrubs to regenerate; crop fields, actively cultivated lands like maize or bean farms; plantation forests, man-made forests dominated by planted species such as pine with structured but less diverse vegetation; near-natural forests, minimally disturbed areas maintaining native vegetation and high biodiversity that support diverse wildlife; and bushes, semiopen shrub- and grass-dominated zones serving as transitional areas and foraging grounds for small mammals. The households were selected using a simple random sampling technique from the list of households provided by village leaders. Verbal consent was sought from the household owners (either of the parents) prior to data collection.

### 2.3. Trapping and Identification of Small Mammals

Small mammals were collected using Sherman live traps baited with a mixture of peanut butter and maize flour/roasted sardines. Sixty Sherman live traps were placed on the desired outdoor habitat type overnight, whereas only three traps were deployed per household (indoor). In outdoor habitats, six-trap lines were used with 10 traps on each line. Traps were set at an interval of 5 m apart from the trap station and trap lines. Trapping across the study villages was done for 3 consecutive days making it 6 days of sampling per ward. The identification of rodents and shrews as well as the collection of their associated ectoparasites was done in the field. Other measurements were recorded such as weight, tail length, body length, ear length, pes length, and reproductive status to aid the identification of small mammals. Rodents and shrews were anesthetized, one by one, inside a jar with cotton wool soaked in 98% diethyl ether [21, 51, 52] followed by their identification and collection of ectoparasites. After euthanasia, individuals from the bags were disposed of accordingly. The rodents and shrews were identified mostly to species level using morphological keys [53, 54] and with the assistance of experienced personnel at the Institute of Pest Management, Sokoine University of Agriculture, Tanzania.

### 2.4. Collection and Morphological Identification of Fleas

Fleas and other ectoparasites were collected by combing rodents and shrews using a fine shoe brush. The collected fleas, ticks, lice, and mites were preserved in vials with 70% ethanol while awaiting further laboratory analyses. Fleas were processed using slightly modified procedures described by Kilonzo [55]; the preserved flea species were cleared by placing them in 10% potassium hydroxide for 24 h, thereafter washed in distilled water and transferred through serial solutions of ethanol (70%, 80%, 95%, and 100%) for 30 min each. Then, the fleas were transferred to methyl salicylate for 20 min, then placed in xylene for 1 h, thereafter mounted on microscope slides by Canada balsam and identified to genus or species level using morphological keys in Segerman [56] and Kilonzo [55] and examined under a compound microscope. Seven specimens of the most collected flea species (unmounted specimens) *Ctenophthalmus* sp. and *X. cheopis*, one of the most efficient flea vectors, were selected randomly from the study plague-endemic areas and submitted for advanced molecular identification and phylogeny.

### 2.5. Molecular Identification of Fleas

#### 2.5.1. DNA Extraction

Genomic DNA extraction of the individual fleas was done using Quick-DNA Miniprep Plus Kit (Zymo Research) according to the manufacturer's protocol. After extraction, the eluted DNA was stored at −20°C until PCR analysis.

#### 2.5.2. PCR Amplification

The nuclear ribosomal internal transcribed spacer (ITS1) region of flea DNA was amplified using primer sequences: forward primer (ITS1-F: GTAGGTGAACCTGCGGAAGGATCATT) and reverse primer (NC5-R: GCTGCGTTCTTCATCGACCC). PCR amplification was performed using AccuPower PCR PreMix from Bioneer (Bioneer Corporation, 8-11 Munpyeongseo-ro, Daedeok-gu, Daejeon 306-220, Republic of Korea) on GeneAmp PCR System 9700 (Applied Biosystems). The PCR reaction mixture consisted of 2 *μ*L of extracted DNA, 0.5 *μ*L of forward primer, 0.5 *μ*L of reverse primer, and 17 *μ*L of nuclease-free water in a microtube containing AccuPower PCR PreMix concentrate making a total reaction volume of 20 *μ*L. Cycling conditions consisted of initial denaturation at 95°C for 5 min followed by 40 cycles at 95°C for 40 s, 63°C for 40 s, and 72°C for 1 min. The final extension at 72°C for 5 min was performed to complete the extension.

#### 2.5.3. Gel Electrophoresis of PCR Products

Agarose gel (1.5%) was prepared and used, and 4 *μ*L of each sample was loaded into each well of the gel followed by the loading of 4 *μ*L of 100 bp DNA ladder to the first well in order to indicate the size of any fragments. The voltage was set at 100 V, and electrophoresis was allowed to run for 40 min. The DNA fragments were observed as gray bands against a black background on a Bio-Rad's GelDoc EZ Imaging System.

### 2.6. DNA Sequencing

Seven amplicons from samples with good PCR amplification were sent to Macrogen Europe (Meibergdreef 57, 1105 BA, Amsterdam, the Netherlands) for sequencing. The PCR products were purified and sequenced directly using the BigDye Terminator Cycle Sequencing Kit (Applied Biosystems, Foster City, California, United States) and a genetic analyzer (ABI 3730xl System from Applied Biosystems). The raw sequence data were cleaned, edited, and assembled by Geneious prime (Version 2021.2.2) software to obtain consensus sequences.

### 2.7. Data Analysis

Raw data were cleaned and entered on Microsoft Excel 2007. Descriptive data were summarized through tables and graphs. All data were not normally distributed when tested for normality (Kolmogorov–Smirnov test *p* = 0.000). Flea diversity was deduced using the “Shannon–Wiener Index [57],” which is defined as follows: *H*′ (*S*) = −*Σ* pi ln pi, where Pi = ni/*N*, ni = number of individuals of a species at a time *i*, *N* = size of the whole community, *Σ* = number of species, and *S* = total number of species. The prevalence and abundance of fleas were following Bush et al. [58]. The prevalence of flea infestation (the prevalence of flea infestation was defined as the proportion of hosts infested with at least one flea relative to the total number of hosts examined) was compared across habitats, species, and sex of rodent hosts in cross-tabulation by chi-square test.

The relationship between abundance of flea species, including the total flea abundance (calculated as the mean number of fleas per individual host, including both infested and noninfested hosts) and the abundance of dominant flea species and various fixed factors (independent factors), namely, habitat type, host species, host sex, and wards, was established by using generalized linear models (GLMs). The negative binomial distribution with a log link function was used in the model to account for overdispersion in the flea count data, where the variance exceeded the mean. Different models were fitted separately to explore the influence of the aforementioned factors on the total flea abundance and the abundance of the four most dominant flea species. In all models, the body length was used as a covariate, which is a proxy for body mass; in addition, all fixed effects were included as a main effect [43]. The initial model included all the factors (host species, host sex, host age, habitat type, and wards); then, nonsignificant factors (*p* > 0.05) were dropped until the stable model with the lowest Akaike information criterion was obtained. A backward stepwise procedure was applied to retain only significant predictors in the final model, ensuring robust identification of factors associated with flea prevalence and abundance. Body length was not significant (*p* > 0.05); hence, we report the GLM results without body length. The prevalence and abundance of the most abundant species were included in the model. IBM SPSS Version 23 was used for all statistical analyses, and a *p* value of less than 0.05 was considered significant. In addition, R Version 4.2.1 statistical software was used to produce figures.

### 2.8. Phylogenetic Analysis

The nucleotide sequences were subjected to basic local alignment search tool (BLAST) to compare their identity with flea species available in nucleotide databases (DDBJ, EMBL, and GenBank databases). The alignment of partial nucleotide sequences of the ITS1 region from this study, with selected closely related reference ITS1 nucleotide sequences from nucleotide databases, was performed using ClustalW in MEGA 11 software. The phylogenetic analysis was done using the maximum likelihood method, utilizing the bootstrap test method with 1000 replicates which is included in MEGA 11. The phylogenetic tree was generated based on partial nucleotide sequences of the flea ITS1 region from 6 samples and 10 nucleotide sequences from reference strains obtained from the nucleotide databases (DDBJ, EMBL, and GenBank) with accession numbers as indicated in Table 1.

## 3. Results

### 3.1. Small Mammals and Flea Species Composition

**Figure 2:**
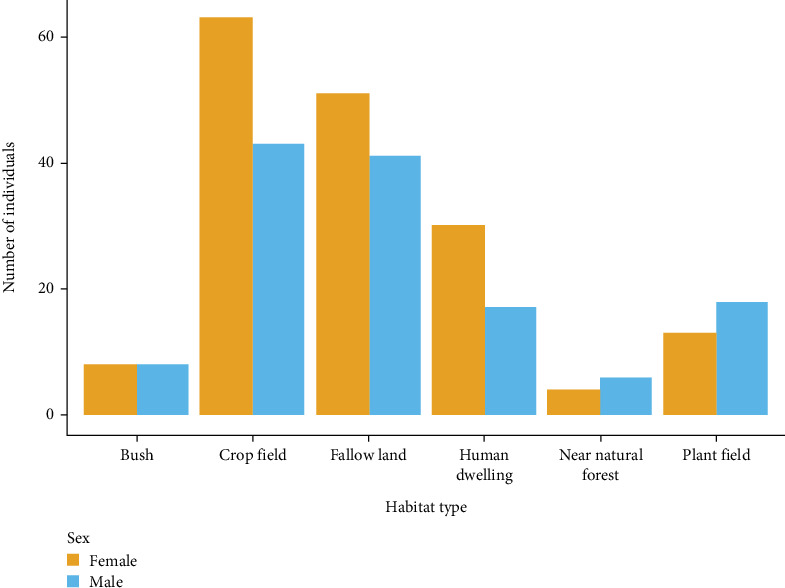
Total count of rodents and shrews per sex captured in different habitats across the study areas in Tanzania, 2022.

A total of 302 small mammals, belonging to 10 genera, were captured during the study period including 271 (89.7%) rodents and 31 (10.3%) shrews. The three most predominant species were *M. natalensis* (*n* = 163, 54.0%), *R. rattus* (*n* = 41, 13.6%), and *Crocidura* spp. (*n* = 31, 10.3%). Other less abundant species captured were *Praomys delectorum* (*n* = 13, 4.3%), *Lophuromys flavopunctatus* (*n* = 13, 4.3%), *Lophuromys kilonzoi* (*n* = 10, 3.3%), *Grammomys* sp. (*n* = 9, 3.3%), *Arvicanthis nairobae* (*n* = 7, 2.3%), *Mus* spp. (*n* = 7, 2.3%), *Acomys* sp. (*n* = 4, 1.3%), *Lemniscomys striatum* (*n* = 3, 1.0%), and *Acomys wilsoni* (*n* = 1, 0.3%). Most of them were captured from the fallow lands (*n* = 104, 34.4%), crop fields (*n* = 94, 31.1%), and human dwellings (*n* = 47, 15.6%). The remaining habitats (plantation forest, bushes, and near-natural forest) altogether consisted of 57 (18.9%) of the captured small mammals irrespective of species. Most of the captured small mammals were females (*n* = 169, 56.0%) rather than males (*n* = 133, 44.0%) in crop fields, fallow land, and human dwellings (Figure 2).

A total of 271 fleas, belonging to nine species, were collected during the study period. The four dominant flea species were *Ctenophthalmus* sp. (*n* = 84, 31.0%) followed by *Pulex irritans* (*n* = 82, 30.3%), *D. lypusus* (*n* = 78, 28.8%), and *Nosopsyllus incisus* (*n* = 11, 4.1%). The less predominant species were *Dinopsylla* sp. (*n* = 5, 1.8%), *X. cheopis* (*n* = 4, 1.5%), *Echidnophaga gallinacea* (*n* = 4, 1.5%), *X. brasiliensis* (*n* = 2, 0.7%), and *Ctenocephalides felis* (*n* = 1, 0.4%) (Table 3). The majority of fleas were collected from the rodent species *M. natalensis* (*n* = 108, 39.9%), *R. rattus* (*n* = 74, 27.3%), *L. flavopunctatus* (*n* = 20, 7.4%), *P. delectorum* (*n* = 26, 9.6%), and *L. kilonzoi* (*n* = 19, 7.0%). The dominant flea species, *Ctenophthalmus* sp., was collected in relatively high numbers from *M. natalensis* (*n* = 21, 7.7%) followed by *L. flavopunctatus* (*n* = 16, 5.9%), *P. delectorum* (*n* = 23, 8.5%), and *L. kilonzoi* (*n* = 17, 6.3%). The next dominant flea species *D. lypusus* was collected in relatively high numbers from *M. natalensis* (*n* = 71, 26.2%), *P. delectorum* (*n* = 3, 1.1%), and *Acomys* sp. (*n* = 2, 0.7%). The human flea (*P. irritans*) was collected in relatively high numbers from *R. rattus* (*n* = 69, 25.5%). No fleas were collected from shrews, *Crocidura* spp., or from the spiny mouse *Acomys wilsoni*. Details for small mammals and flea species composition are shown in Tables 2 and 3.

### 3.2. Flea Diversity

**Figure 3:**
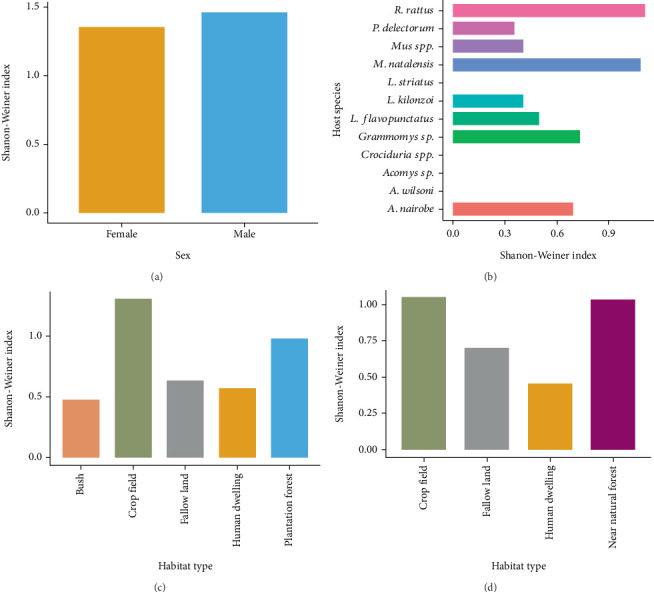
Shannon–Wiener species diversity index values for flea species across. (a) Sex. (b) Host species. (c) Different types of habitats in Lushoto District, Tanzania, 2022. (d) Different types of habitats in Mbulu District.

The highest flea diversity in selected study sites within Lushoto District was recorded in crop fields (*H*′ = 1.3) followed by near-natural forest (*H*′ = 0.972) and fallow land (*H*′ = 0.637). The lowest flea diversity in the area was recorded in human dwellings (*H*′ = 0.572) and bush (*H*′ = 0.472) habitats (Figure 3). Similarly, the highest flea diversity in selected study sites within Mbulu District was recorded in crop fields (*H*′ = 1.05) followed by near-natural forests (*H*′ = 1.03) and fallow lands (*H*′ = 0.7). The lowest diversity was recorded in human dwellings (*H*′ = 0.453, Figure 3). Interestingly, rodent species such as *Grammomys* sp., *A*. *nairobae*, *L. flavopunctatus*, *Mus* spp., *L. kilonzoi*, and *P. delectorum*, which had been infested with a small number of fleas, were relatively richer in terms of flea species (Figure 3). Male rodents had higher flea species diversity relative to females (Figure 3).

### 3.3. Flea Prevalence and Abundance

The overall prevalence of fleas across the study areas was 32.1%, 95% CI [26.9, 37.7]. The overall prevalence did not vary across the different habitat types (*p* = 0.150). Fleas were more prevalent on *M. natalensis* (16.2%), followed by *R. rattus* (5.0%), *L. flavopunctatus* (3.3%), *L. kilonzoi* (2.3%), and *P. delectorum* (1.7%) (Table 4). The highest prevalence of fleas was recorded in crop fields, fallow lands, and human dwellings in that order. Nevertheless, the prevalence of fleas only varied between crop fields/fallow lands relative to human dwellings (*X*^2^ = 4.1568, df = 1, *p* = 0.0415) and near-natural forests (*X*^2^ = 5.139, df = 1, *p* = 0.0234).

**Figure 4:**
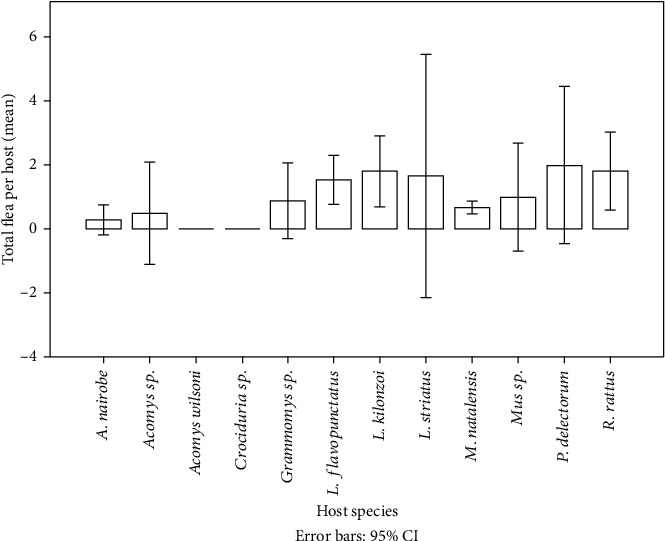
Total number of fleas (mean) on each of the collected small mammal species in both endemic and nonendemic areas.

The total mean abundance in each small mammal host species is shown in Figure 4. The total flea abundance in the study districts varied across habitat types (*p* = 0.001). The most abundant flea species were *Ctenophthalmus* sp. (31.0%) (4.52 ± 0.44), *P. irritans* (30.3%) (9.15 ± 0.77), *D. lypusus* (28.8%) (2.82 ± 0.9), and *N. incisus* (4.1%) (1.73 ± 0.27).

The overall prevalence and the total abundance varied with the sex of the host (*p* < 0.05). The prevalence and total abundance of fleas were relatively higher on male than female rodents (Table 4). Whereas the total abundance of fleas did not vary between individual host species (*p* = 0.275) and wards (*p* = 0.153), the prevalence did vary between wards (Table 4). Furthermore, the mean abundance of *Ctenophthalmus* sp., the most predominant species, varied significantly between host species (*p* = 0.000) and habitats (*p* = 0.002). However, it did not vary significantly between sex (*p* = 0.230) and wards (*p* = 0.680) across the study districts altogether. Conversely, the mean abundance of *D. lypusus* varied significantly between host sex (*p* = 0.011) but not between host species (*p* = 0.471), habitats (*p* = 0.258), and wards (*p* = 0.052). For *P. irritans*, the mean abundance varied significantly between habitats (*p* = 0.001) but not between host species (*p* = 0.919), host sex (*p* = 0.589), and wards (*p* = 0.471).

### 3.4. Phylogeny of Selected Flea Species

The ITS1 nucleotide sequence of *Ctenophthalmus* sp. from Lushoto (AM142/Ctp/Tanzania/2022) showed 99.47% identity with the sequence from Mbulu (AM11/Ctp/Tanzania/2022) obtained in this study. However, the ITS1 nucleotide sequence of *Ctenophthalmus* sp. from Mbulu (AM39/Ctp/Tanzania/2022) showed lower identity when aligned with the other two sequences, with 89.0% similarity to AM11/Ctp/Tanzania/2022 and 89.55% to AM142/Ctp/Tanzania/2022.

**Figure 5:**
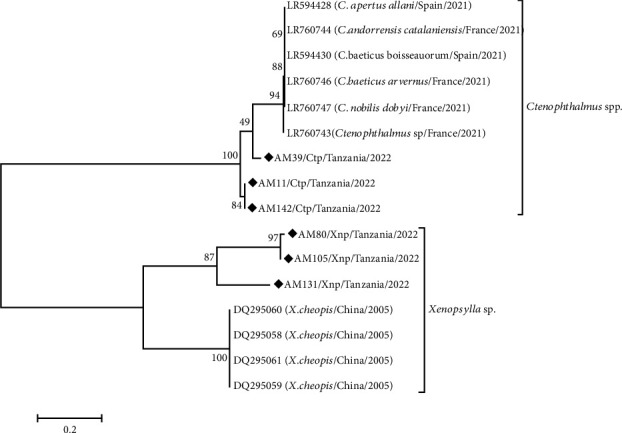
Evolutionary analysis by maximum likelihood method to infer evolutionary history. The tree shown is the one with the highest log likelihood. Next to the branches is the percentage of trees in which the fleas are clustered together. Black rhombus denotes sequences from this study.

Upon multiple alignment of the three sequences from the present study of *Ctenophthalmus* sp., the four ITS1 sequences of isolates from France of 2021 (Accession Numbers LR760743, LR760744, LR760746, and LR760747), and the two ITS1 sequences of *Ctenophthalmus* sp. isolates from Spain of 2021 (Accession Numbers LR594428 and LR594430), the lowest nucleotide identity was 79.74%, and the highest was 82.15%, with a great number of indels (Figure 5).

The ITS1 nucleotide sequences of two *X. cheopis* isolates from this study, one from Lushoto (AM105/Xnp/Tanzania/2022) and another from Mbulu (AM80/Xnp/Tanzania/2022), showed 98.44% identity upon alignment. In contrast, another *X. cheopis* isolate from Lushoto (AM131/Xnp/Tanzania/2022) exhibited numerous indels and lower similarity when aligned with AM80 and AM105, showing nucleotide identities of 73.25% and 73.69%, respectively. Upon multiple alignment of the three sequences from our study of *X. cheopis* and the four ITS1 sequences of isolates from China in 2005 (Accession Numbers DQ295060, DQ295058, DQ295061, and DQ295059), the lowest nucleotide identity was 51.54%, and the highest was 52.40%, with a large number of indels (Figure 5).

## 4. Discussion

Nine flea species were collected, with *Ctenophthalmus* sp., *P. irritans*, *D. lypusus*, and *N. incisus* being most abundant. Endemic areas showed higher flea diversity, while nonendemic areas had only *P. irritans* on *R. rattus*, likely due to climatic differences [11, 21]. High prevalence of *D. lypusus* and *Ctenophthalmus* sp. in endemic regions aligns with previous Tanzanian reports [23, 24, 59–62]. These nonhost-specific fleas are endemic to Lushoto and Mbulu districts [23]. *X. cheopis* and *X. brasiliensis*, key plague vectors in Tanzania [23, 24, 59, 60], yet showed low abundance during this study; this might suggest shifts in species composition and plague dynamics driven by ecological disturbance, rodent changes, climate, and vector control; however, extensive study is needed to get a clear picture [63, 64].

Most fleas collected in this study were from the *Natal multimammate*, a known reservoir in past plague outbreaks in Tanzania and beyond [21, 23, 59, 62]. Its wide habitat distribution, high reproductive rate, and adaptability to environmental changes likely explain its dominance [28, 65]. The strong association of *M. natalensis* with flea vectors and its reported resistance to plague infection highlight its ongoing role in plague and other disease transmission, particularly in endemic areas, while its role in nonendemic areas appears limited due to lower flea abundance and diversity [23, 41, 50].

The absence of fleas on *Crocidura* spp. in our study is likely influenced by multiple factors observed in the field. Most of the captured *Crocidura* individuals were found dead, and as fleas leave dead hosts as soon as body temperature starts to drop, this would have directly reduced the likelihood of detecting ectoparasites [65]. Additionally, the relatively low numbers of *Crocidura* captured may have limited the chances of encountering infested individuals [66]. Certain species of *Crocidura* may also not be preferential hosts for fleas due to their grooming behavior, the smell produced to mark their territory, and their insectivorous diet, which reduces contact with flea-infested environments [67]. While it is possible that live *Crocidura* could host fleas, the combination of low abundance, dead hosts, host-specific traits, and dietary habits likely explains the observed absence, consistent with ecological conditions in the study areas [23, 41, 68, 69].

The highest flea diversity index in crop fields and near-natural forests for both Lushoto and Mbulu districts is consistent with previous studies [23, 41]. This elevated diversity may be attributed to favorable microclimates and high rodent species richness in these habitats, which provide suitable conditions for flea survival and reproduction [23, 28, 41]. High host species richness allows fleas to exploit multiple hosts, increasing vector survival and contact rates, while stable microclimatic conditions enhance flea longevity. Together, these factors facilitate the maintenance of flea populations and can promote the persistence of plague in these endemic areas by sustaining continuous opportunities for pathogen transmission among hosts. Previous studies have shown a positive correlation between flea diversity and host species richness [5, 8, 70]. Additionally, crop fields provide abundant food, supporting rodent populations and attracting hematophagous ectoparasites like fleas. Flea diversity in plague-endemic areas may contribute to the maintenance and persistence of plague in active foci, potentially leading to outbreaks [26, 41, 62]. The lowest flea diversity was observed in human dwellings, likely because dominant rodents like *M. natalensis* and *R. rattus* were mainly infested with *P. irritans*, a flea closely associated with humans. As *P. irritans* primarily parasitizes humans, only a single flea species was found indoors, unlike other habitats with multiple species [20].

Flea prevalence and abundance varied significantly across habitats, with the highest levels in crop fields and fallow land. This variation likely reflects differences in host availability, particularly between generalist and specialist species. Generalists like *M. natalensis* typically harbor more ectoparasites than specialists such as *Graphiurus murinus* [28]. It is well known, from several studies, that environmental factors, including temperature and humidity gradients, also influence flea distribution although these factors were not measured in this study [23, 71]. As flea development depends on environmental conditions, habitats with optimal temperature and humidity favor larval survival and metamorphosis [24, 25, 35].

Flea prevalence, abundance, and diversity were generally higher on male rodents, consistent with male-biased parasitism [40, 70]. This may result from larger body size, increased surface area, and higher exposure due to exploratory behavior and wider home ranges [3, 28, 39]. Hormonal differences, particularly testosterone-linked immunosuppression, also reduce male resistance to infestation [42, 47]. Grooming behavior further influences infestation, with less grooming associated with higher flea loads [48]. Older rodents often carry more fleas due to increased exposure over time and greater nutritional resources for ectoparasites [23, 31, 39].

Flea infestation was higher in plague-endemic wards than in nonendemic areas, likely due to favorable environmental conditions, host diversity, and factors like age, sex, and immunity. Similar trends have been reported in Tanzania and other regions [23, 24, 41, 72]. Elevated flea abundance increases the risk of convergence between enzootic and epizootic plague cycles [25], and the diversity of fleas and small mammals enhances their interaction, promoting plague persistence in active foci [24].

In this study, phylogenetic analysis of the ITS1 sequence of *Ctenophthalmus* sp. from Lushoto (AM/142/Ctp/Tanzania/2022) showed high nucleotide identity with *Ctenophthalmus* sp. from Mbulu (AM/11/Ctp/Tanzania/2022). Similarly, *X. cheopis* from Lushoto (AM/105/Xnp/Tanzania/2022) was closely related to *X. cheopis* from Mbulu (AM/80/Xnp/Tanzania/2022). This high similarity may reflect the historical introduction of fleas from Lushoto to Mbulu during early plague outbreaks via human movement, contributing to both areas becoming endemic foci [16].

The lower similarity between *Ctenophthalmus* sp. from this study and sequences from Spain and France likely reflects the genus's high genetic diversity, with many species and subspecies, only a fraction of which are represented in public databases [4, 41, 73]. In contrast, the low similarity between Tanzanian *X. cheopis* and Chinese isolates may result from high intraspecific ITS1 variation driven by geographic and environmental differences, leading to genetic divergence through adaptation. Similar variation was reported among *X. cheopis* populations in Madagascar and Mayotte [73]. It is also possible that the Tanzanian isolates represent new subspecies of *Xenopsylla* sp. that share a recent common ancestor. Confirmation would require further studies using alternative genetic markers, such as mitochondrial genome analysis, which offers deeper insights into flea taxonomy and phylogeny [74]. Multilocus sequence analysis may also help clarify their taxonomic status.

## 5. Conclusions

This study demonstrates that flea diversity, prevalence, and abundance vary across rodent host species and habitats, with *Ctenophthalmus* sp., *P. irritans*, *D. lypusus*, and *N. incisus* being the most prevalent and abundant. Habitats such as crop fields, fallow land, and near-forest areas support higher flea diversity and abundance, contributing to conditions favorable for plague persistence. Phylogenetic analysis of ITS1 sequences for *Ctenophthalmus* sp. and *X. cheopis* revealed strong nucleotide identity, providing new insights into vector relationships in Tanzania. These findings underscore the need for regular surveillance of flea vectors and rodent hosts in plague-endemic areas to inform control strategies. Future studies should focus on longitudinal monitoring, molecular screening of fleas and hosts, and the role of ecological and climatic factors in shaping flea–host dynamics.

## Figures and Tables

**Table 1:** BLAST results of flea sequences showing matching species with ITS1 targeted marker, base pairs, country of origin, and accession number.

**Our sample sequences**		**BLAST results**		
	**Base pairs**	**Matching species**	**Country of origin**	**Accession number**
*Ctenophthalmus* sp. (AM11/Ctp/Tanzania/2022; AM142/Ctp/Tanzania/2022; AM39/Ctp/Tanzania/2022)	888	*Ctenophthalmus* sp.	France	LR760743
	888	*Ctenophthalmus andorrensis catalaniensis*	France	LR760744
	888	*Ctenophthalmus baeticus arvernus*	France	LR760746
	888	*Ctenophthalmus nobilis dobyi*	France	LR760747
	888	*Ctenophthalmus apertus allani*	Spain	LR594428
	889	*Ctenophthalmus baeticus boisseauorum*	Spain	LR594430

*Xenopsylla cheopis* (AM80/Xnp/Tanzania/2022; AM105/Xnp/Tanzania/2022; AM131/Xnp/Tanzania/2022)	1484	*Xenopsylla cheopis*	China	DQ295060
	1484	*Xenopsylla cheopis*	China	DQ295058
	1484	*Xenopsylla cheopis*	China	DQ295059
	1484	*Xenopsylla cheopis*	China	DQ295061

**Table 2:** Small mammals and their associated abundant fleas in sampled plague-endemic and nonendemic areas in Tanzania, 2022.

**Flea species**	**Host species from each district**		
	**Lushoto District**	**Mbulu District**	**Morogoro Rural District**
*Ctenophthalmus* sp.	*A. nairobae, Grammomys* sp., *L. kilonzoi*, *M. natalensis*, and *P. delectorum*	*L. flavopunctatus*, *L. striatus*, and *M. natalensis*	None
*Pulex irritans*	*M. natalensis* and *R. rattus*	*M. natalensis*, *Mus* spp. and *R. rattus*	*R. rattus*
*Dinopsylla lypusus*	*L. kilonzoi*, *M. natalensis*, *Mus* spp., and *P. delectorum*	*Acomys* sp., *M. natalensis*, and *P. delectorum*	None
*Nosopsyllus incisus*	*Grammomys* sp. and *M. natalensis*	*L. flavopunctatus*	None

**Table 3:** Flea counts and mean ± SEM in rodents and shrews collected in selected areas of Mbulu, Lushoto, and Morogoro Rural districts, Tanzania, from March to June 2022.

**Host species**	**No. hosts**	**Total no. fleas collected per host (no. fleas/host examined) and ** **m** **e** **a** **n** ± **SEM**									
		***C*s**	***Dl***	***D*s**	***Xb***	***Xc***	***Cf***	***Eg***	***Ni***	***Pi***	**Total**
*M. natalensis*	163	21 (2 ± 0)	71 (3 ± 0)	3 (1 ± 0)	2 (2 ± 0)	3 (2 ± 0)	0	0	1	7 (2 ± 0)	108
*R. rattus*	41	0	0	0	0	0	1	4 (4 ± 0)	0	69 (10 ± 1)	74
*Crocidura* spp.	31	0	0	0	0	0	0	0	0	0	0
*L. flavopunctatus*	13	16 (2 ± 0)	0	0	0	0	0	0	4 (1 ± 0)	0	20
*P. delectorum*	13	23 (11 ± 0)	3 (2 ± 0)	0	0	0	0	0	0	0	26
*L. kilonzoi*	10	17 (3 ± 0)	1	1	0	0	0	0	0	0	19
*Grammomys* sp.	9	1	0	0	0	1	0	0	6 (2 ± 0)	0	8
*A. nairobae*	7	1	0	1	0	0	0	0	0	0	2
*Mus* spp.	7	0	1	0	0	0	0	0	0	6 (4 ± 1)	7
*Acomys* sp.	4	0	2 (2 ± 0)	0	0	0	0	0	0	0	2
*L. striatus*	3	5 (3 ± 0)	0	0	0	0	0	0	0	0	5
*A. wilsoni*	1	0	0	0	0	0	0	0	0	0	0
Total	302	84	78	5	2	4	1	4	11	82	271

Abbreviations: *Cf*, *Ctenocephalides felis*; *C*s, *Ctenophthalmus* sp.; *Dl*, *Dinopsylla lypusus*; *D*s, *Dinopsylla* sp.; *Eg*, *Echidnophaga gallinacea*; *Ni*, *Nosopsyllus incisus*; *Pi*, *Pulex irritans*; *Xb*, *Xenopsylla brasiliensis*; *Xc*, *Xenopsylla cheopis*.

**Table 4:** Prevalence of flea species with respect to sex, habitat, and wards of host species collected in selected areas of Mbulu, Lushoto, and Morogoro Rural districts, Tanzania, from March to June 2022.

**Variable**	**Categories**	**Total number**	**Infested**	**Not infested**	**Prevalence (%)**	**p** ** value**
Sex	Female	169	46	123	15.2	0.040^∗^
	Male	133	51	82	16.9	

Habitat	Crop field	94	28	66	9.3	0.150
	Fallow land	104	27	77	8.9	
	Human dwellings	47	20	27	6.6	
	Plantation forest	31	11	20	3.6	
	Near-natural forest	10	6	4	2.0	
	Bush	16	5	11	1.7	

Wards	Bwakila Chini	59	0	59	0.0	0.000^∗^
	Kisaki	22	3	19	1.0	
	Manolo	43	16	27	5.3	
	Nahasey	55	22	33	7.3	
	Shume	73	26	47	8.6	
	Yaeda Ampa	50	30	20	9.9	

Host species	*M. natalensis*	163	49	114	16.2	0.000^∗^
	*R. rattus*	41	15	26	5.0	
	*Crocidura* spp.	31	0	31	0.0	
	*L. flavopunctatus*	13	10	3	3.3	
	*P. delectorum*	13	5	8	1.7	
	*L. kilonzoi*	10	7	3	2.3	
	*Grammomys* sp.	9	3	6	1.0	
	*A. nairobae*	7	2	5	0.7	
	*Mus* spp.	7	3	4	1.0	
	*Acomys* sp.	4	1	3	0.3	
	*L. striatus*	3	2	1	0.7	
	*A. wilsoni*	1	0	1	0.0	

⁣^∗^Statistically different.

## Data Availability

Datasets from this study can be provided upon reasonable request.
